# Enhanced IgG_1_‐mediated antibody response towards thymus‐dependent immunization in CXCR1‐deficient mice

**DOI:** 10.1002/iid3.380

**Published:** 2020-11-23

**Authors:** Jennifer Jaufmann, Melanie Carevic, Leyla Tümen, Derya Eliacik, Fee Schmitt, Dominik Hartl, Sandra Beer‐Hammer

**Affiliations:** ^1^ Department of Pharmacology, Experimental Therapy, and Toxicology, Institute of Experimental and Clinical Pharmacology and Pharmacogenomik and ICePhA University of Tuebingen Tuebingen Germany; ^2^ Children's Hospital and Interdisciplinary Center for Infectious Diseases University of Tuebingen Tuebingen Germany; ^3^ Novartis Institutes for Biomedical Research, Novartis Campus Basel Switzerland

**Keywords:** B‐1 cells, B‐2 cells, chemokine receptors, CXCR1, germinal center reaction, innate and adaptive antibody responses, vaccination

## Abstract

**Background:**

Chemokine receptors and their corresponding ligands are key players of immunity by regulation of immune cell differentiation and migration. CXCR1 is a high‐affinity receptor for CXCL8. Differential expression of CXCR1 is associated with a variety of human pathologies including cancer and inflammatory diseases. While various studies have highlighted the importance of CXCR1‐mediated CXCL8‐sensing for neutrophil trafficking and function, its role in B‐cell responses remains unsolved. Therefore, our aim was to investigate innate and adaptive antibody responses in CXCR1‐deficient mice.

**Methods:**

Cell populations of the spleen and the peritoneal cavity were identified and quantified via flow cytometry. To investigate thymus‐independent (TI) and thymus‐dependent (TD) antibody responses, mice were immunized intraperitoneally with TNP‐Ficoll, Pneumovax23, and TNP‐Chicken Gamma Globulin. Mice were bled before as well as 7 and 14 days after vaccination to collect serum. Serum antibody levels overtime were analyzed according to their specificity by enzyme‐linked immunosorbent assay. B‐1 cell functionality was examined by IL‐5/IL‐5Rα‐dependent stimulation of peritoneal and splenic cells in vitro. To analyze CXCR1/2‐expression, CD19^+^ splenocytes were enriched by magnetic‐activated cell sorting before isolation of total RNA contents, followed by reverse transcription and real‐time polymerase chain reaction.

**Results:**

The distribution of natural B‐1 cell populations was disturbed in the absence of CXCR1, while their responsiveness towards TI antigens and in vitro stimulation remained functional. Besides, CXCR1‐deficiency was accompanied by increased frequencies of follicular B‐2 cells in the spleen. Interestingly, these mice produced elevated levels of antigen‐specific IgG_1_ upon TD immunization and harbored a significantly enlarged proportion of CXCR5‐expressing T helper (H) cells. CXCR1‐expression was detectable in CD19^+^ splenocytes derived from wild‐type, but not CXCR1‐deficient mice.

**Conclusion:**

Our data demonstrate a previously unknown relevance of CXCR1 for the production of specific IgG_1_ in response to vaccination. These findings identify CXCR1 as a promising candidate for future studies on the regulation of adaptive antibody responses.

## Graphical abstract

The spatial distribution of natural B‐1 cells was disturbed in the absence of CXCR1, while their responsiveness towards thymus‐independent antigens and in vitro stimulation remained functional. Moreover, CXCR1‐deficiency was accompanied by greatly increased numbers and frequencies of follicular B‐2 cells in the spleen. Interestingly, these mice produced elevated levels of antigen‐specific IgG_1_ upon thymus‐dependent immunization and harbored significantly increased populations of GLY7^+^ germinal center (GC) B cells and CXCR5^+^ T follicular helper (FH) cells.

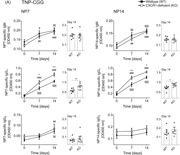

## 1 INTRODUCTION

Chemokines are a large family of cytokines initially described as chemotactic molecules, since they are key regulators of immune cell positioning and lymphocyte trafficking. They execute their function by binding to respective chemokine receptors, which mainly belong to the group of G‐protein‐coupled receptors (GPCRs).^1^ Beyond attracting immune cells towards sites of infection and inflammation, chemokines also contribute to embryonic development, regulate angiogenesis and promote proliferation, and metastasis of tumor cells.^2^, ^3^ Thus, chemokines and their receptors are potential candidates for the development of novel immunotherapeutic approaches.

Human CXCR1 and CXCR2 are high affinity receptors for the CXC‐chemokine ligand 8 (CXCL8/interleukin‐8 [IL‐8]). Both of them are highly expressed on the surface of neutrophils, but also on macrophages, T cells and normal as well as malignantly transformed B cells.^4^, ^5^, ^6^ In humans, CXCL8 is rapidly produced under inflammatory conditions and leads to the attraction of neutrophils into inflamed microenvironments, such as the airways, where they get activated and provide defense against bacterial pathogens.^7^, ^8^

Whereas CXCR2 mainly mediates neutrophil transmigration, studies suggest an important role for CXCR1 in the course of bacterial sensing and killing. In 2014, Carevic et al.^9^ reported reduced Toll‐like receptor (TLR) 5‐expression and impaired reactive oxygen species‐production of neutrophils during *Pseudomonas aeruginosa* infection in CXCR1‐deficient mice. In humans, CXCR1 gene variants and expression levels have been shown to correlate with cystic fibrosis lung disease and proteolytic cleavage of CXCR1 impaired antibacterial neutrophil host defense functions.^7^, ^10^ In summary, these studies suggest an essential role of CXCR1 in airway infection and innate immunity.

While the role of CXCR1 for neutrophil function is subject of various studies, its role for B‐cell biology remains largely unknown. B cells are essential mediators of both innate and adaptive immunity by providing humoral protection.^11^ Generally, they can be divided into conventional B‐2 and innate‐like B‐cell populations, with the latter comprising natural B‐1 and marginal zone (MZ) B cells.

Thymus‐independent (TI) antigens such as pneumococcal polysaccharides (pPS) rapidly trigger the activation of innate‐like B cells without the need for T‐cell help, for example, via B‐cell receptor crosslinking and TLR‐engagement.^12^ This response results in short‐lived plasma cell differentiation, accompanied by low levels of somatic hypermutation and inducing antibodies of rather broad specificity.^12^, ^13^ Innate B cells are characterized by a unique expression of surface markers, the ability of self‐replenishment and their specific anatomical localization. In mice, MZ B cells are restricted to the spleen where they interact with blood‐borne antigens and produce protective immune globulin (Ig).^14^, ^15^ By contrast, natural B‐1 cells are enriched within the pleural and peritoneal body cavities and can also be found in the spleen.^16^ Under homeostatic conditions, B‐1 cell populations are maintained by self‐renewal and constitutively secrete autoreactive antibodies, clearing apoptotic cells and toxic metabolites. Besides, they respond towards common danger‐associated epitopes, thereby substantially contributing to the clearance of encapsulated bacteria.^17^, ^18^, ^19^, ^20^, ^21^, ^22^

On the other hand, long‐lived adaptive B‐cell responses towards thymus‐dependent (TD) protein antigens are usually driven by follicular (FO) B‐2 cells in cooperation with cognate T helper (T_H_) cells during a process called germinal center (GC) reaction. It comprises several rounds of affinity maturation and selection, eventually resulting in highly specific and class‐switched effector B cells.^23^ Meanwhile, the function of T cells is to provide signals essential to the proliferation, survival, and differentiation of maturing B cells. GC formation is a dynamic process that critically depends on coordinated changes in chemokine receptor expression, for instance affecting CXCR4, CXCR5, and CCR7.^24^ Tight regulation of these processes is crucial to maintain self‐tolerance and aberrant adaptive antibody responses are associated with severe autoimmune pathologies in humans.^25^, ^26^

In the present study, we analyzed innate and adaptive B‐cell populations in the absence of CXCR1 in mice. Concomitantly, we evaluated in vivo antibody responses to immunization with TI and TD antigens, considering different Ig isotypes.

While frequencies of FO B cells were enhanced in the spleen of CXCR1‐deficient mice, the population of innate‐like B‐1 cells was significantly reduced. CXCR1‐deficiency favored the production of specific serum IgG_1_ towards the TD antigen TNP‐CGG, accompanied by increased percentages of CXCR5‐expressing T_H_ cells.

In conclusion, our results reveal a novel role of CXCR1 during the formation of TD antibody responses.

## 2 MATERIALS AND METHODS

### 2.1 Mice

CXCR1‐deficient mice were generated by P. Murphy/NIH as described previously and purchased from The Jackson Laboratory (strain B6.129‐*Cxcr1* ^*tm1Msli*^/J).^9^ Mice were bred at the animal facility of the Institute of Pharmacology and Toxicology in Tübingen under specific pathogen‐free conditions in open cages. For all experiments, age‐ and sex‐matched littermate controls were used (10–15 weeks old). All animal work was performed according to the German animal care regulations and animal experiments were approved by the local ethics committee (AZ 04.01.2018; PH2/18).

### 2.2 Organ preparation and cell culture

Peritoneal cells were harvested via lavage with 10 ml of ice cold PBS. Spleens were isolated and homogenized using a 70‐µm cell strainer, followed by erythrocyte lysis for 3 min to get rid of red blood cells. Subsequently, single cell suspensions were analyzed by flow cytometry either directly or after in vitro stimulation.

For in vitro assays, 2 × 10^6^ cells were cultured in 500‐µl cell culture medium (RPMI 1640 supplemented with 10% FCS, 1% l‐glutamine, 1% penicillin/streptomycin, and 0.05‐mM β‐mercaptoethanol) in 24‐well plates. For stimulation, 10‐ng/ml IL‐4, 2‐µg/ml α‐CD40, and 10‐ng/ml IL‐5 were supplemented and cells were incubated for 48 h at 37°C and 5% CO_2_.

### 2.3 Flow cytometry

To investigate B‐1 cell populations pre‐ or poststimulation, splenocytes and peritoneal cells were stained with CD19‐V450, CD43‐PE, CD5‐APC, IgM‐PE‐Cy7, and CD125‐FITC. For analysis of B‐1 cell intracellular IgM‐expression after stimulation, cells were initially stained on the surface with CD19‐V450, CD43‐FITC, CD5‐APC, and IgM‐PE. Fixation, permeabilization, and intracellular staining were performed using the Transcription Buffer Set (BD Bioscience) and IgM‐PE‐Cy7 antibody. To analyze splenic GC B cells and T_FH_ cells, splenocytes were stained with B220‐PerCP, CD21‐FITC, CD23‐BV510 and GLY7‐PE, or CD3‐FITC, CD4‐PacificBlue, CXCR5‐APC and GLY7‐PE, respectively. MZ and FO B cells were stained using B220‐PerCP, CD23‐BV510, CD21‐FITC, IgM‐APC‐Cy7, and IgD‐V450. All fluorchrome‐conjugated antibodies were purchased from BD Bioscience or Biolegend. Before surface staining, unspecific F_C_‐binding sites were blocked by incubation with anti‐CD16/32 (Biolegend) for 15 min. Measurements were performed at the BD FACSCanto II and gating was done using FlowJo Version 10. To ensure correct gating, fluorescence minus one controls were applied.

### 2.4 Immunization protocol

Mice were immunized intraperitoneally with either 1‐µg Pneumovax23 (P23; SanofiPasteurMSD) in 100‐µl PBS, 100‐µg TNP‐CGG (Biosearch Technologies) precipitated in 100‐µl Alum + PBS (1:1) or 5‐µg TNP‐Ficoll (Biosearch Technologies) in 100‐µl PBS. Blood sampling was performed before, as well as 7 and 14 days after immunization. Blood was collected in Microtainer® SST™ Tubes (BD Bioscience) and kept at room temperature for 30–60 min. Subsequently, the serum was separated by centrifugation (90 s at 15,000 g) and stored at −20°C.

### 2.5 Enzyme‐linked immunosorbent assay

To measure concentrations of global serum IgM, high‐binding 96‐well plates were coated with purified rat anti‐mouse IgM capture antibodies (BD Pharmingen) at 4°C over night. The other day, the plate was blocked for 1 h with blocking buffer. Diluted sera were incubated on precoated plates for 2 h at room temperature. Purified mouse ĸ IgM isotype control (BD Pharmingen) was used as a standard in serial dilutions.

For detection of P23‐ and pPS‐specific antibodies in the sera of mice, high‐binding 96‐well plates were coated over night at 37°C with 1‐µg/ml P23, pPS3, 4, 6B, or 19F (SSI Diagnostica). Antibody responses towards TNP‐Ficoll/‐CGG were evaluated by coating of 10‐µg/ml NP7‐BSA or NP14‐BSA (Biosearch Technologies) at 4°C over night. Sera were diluted in sample buffer and P23‐probes were freshly supplemented with 10‐µg/ml cell wall polysaccharides (SSI Diagnostica) to capture unspecific antibodies. Serum dilutions were incubated on precoated, blocked plates for 2 h at 37°C (P23 and pPS) or for 1 h at room temperature (TNP‐Ficoll/CGG).

Specifically bound antibodies were detected by incubation with biotin‐conjugated anti‐mouse IgM, IgG_1_, IgG_2a_, or IgG_3_ (BD Pharmingen).

For all enzyme‐linked immunosorbent assays (ELISAs) subsequently, streptavidin‐conjugated horseradish peroxidase (Bio‐Techne) was incubated and chemoluminescent reaction was induced by addition of 3,3′,5,5′‐tetramethylbenzidine substrate (Thermo Fisher Scientific). The reaction was stopped with sulfuric acid and measurement was performed at a wavelength of 450 nm. To allow the direct comparison of samples without specific standard, all probes were measured on the same ELISA plate.

#### 2.5.1 Real‐time polymerase chain reaction

CD19^+^ B cells were isolated from the pool of total splenocytes by performance of magnetic‐activated cell sorting using anti‐CD19 micro beads and magnetic separation columns (Miltenyi Biotech) for positive selection. Subsequently, RNA‐contents were isolated with the ExtractMe Total RNA Kit (Blirt). Four hundred nanograms of RNA were transcribed using TranscriptMe cDNA Kit (Blirt). Subsequently, real‐time polymerase chain reaction (RT‐PCR) was performed with the Sensi‐fast SYBR No‐Rox Kit (Bioline) in the Light Cycler® 480 (Roche). The final amount of cDNA was 50 ng/plate well. CXCR1 and CXCR2 primers were used as previously described by Fu et al.^27^ and produced by Biomers (CXCR1 forward: 5′‐GCTGCCCACTGGAGATTATTTC‐3′, CXCR1 reverse 5′‐TATGCCTGGCGGAAGATAGC‐3′; CXCR2 forward: 5′‐ATGCTGTTCTGCTACGGG‐3′; CXCR2 reverse: 5′‐ATGGATGATGGGGTTAAG‐3′). Amplification was performed under following conditions: preincubation at 94°C (2 min), followed by 30 cycles of each 94°C (30 s), 55°C (30 s), and 72°C (1 min). Advanced relative quantification was done with the Light Cycler® 480 software and β‐actin served as reference gene.

## 3 RESULTS

### 3.1 Skewed repertoire of B‐cell populations in CXCR1‐deficient mice

**Figure 1:**
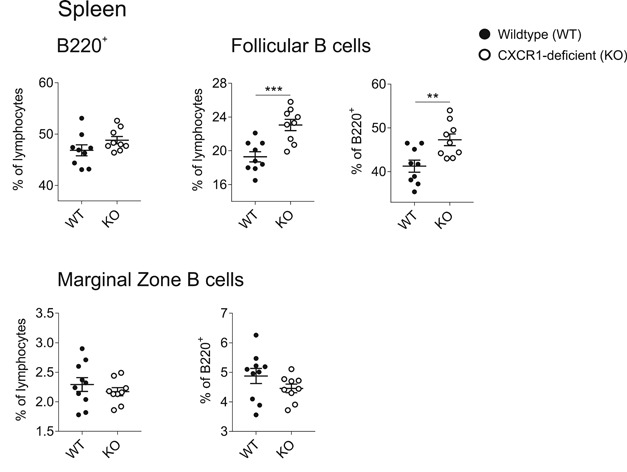
Enriched proportions of follicular (FO) B cells in the spleen of CXCR1‐deficient mice. Splenic single cell suspensions were fluorescently stained with B220‐PerCP, CD23‐BV510, CD21‐FITC, IgM‐APC‐Cy7, and IgD‐V450 and subsequently analyzed by flow cytometry. FO B cells were defined as CD23^+^CD21^low^IgD^+^IgM^low^ and marginal zone B cells as CD23^−^CD21^high^IgM^+^IgD^low^. Cell populations are shown as percentage of all single lymphocytes and percentage within the fraction of B220^+^ cells for wild‐type (WT) and CXCR1‐deficient (KO) mice. Data represent *n* = 9–10 mice per genotype from two independently performed experiments and error bars indicate mean ± *SEM*. Significances were determined by Student's *t*‐test and a *p* < .05 was considered statistically significant (*p <.05, **p < .01, ***p < .001)

Initially, we investigated splenic B‐2 cell populations in CXCR1‐deficient mice as compared to wild‐type (WT) littermate controls. While the number of splenocytes, the frequency of B220^+^ cells and MZ B cells were comparable between the genotypes, the proportion of FO B cells was significantly increased in the context of CXCR1 deficiency (Figure 1).

**Figure 2:**
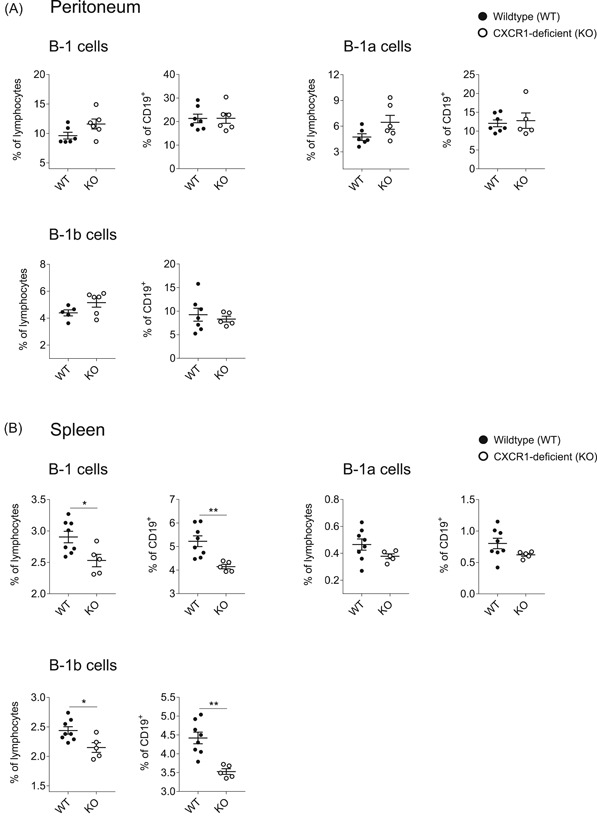
Decreased populations of splenic B‐1 cells in CXCR1‐deficient mice. Cells were fluorescently stained with CD19‐V450, CD43‐PE, CD5‐APC, and IgM‐PE‐Cy7 and subsequently analyzed by flow cytometry. B‐1 cells were defined as CD19^+^CD43^+^IgM^+^ and subdivided into B‐1a and B‐1b cells, being CD5^+^ or CD5^−^, respectively. B‐1 cell frequencies were assessed for wild‐type (WT) and CXCR1‐deficient (KO) mice in (A) peritoneal washouts and (B) the spleen and are given as both, percentage of all single lymphocytes and percentage within the CD19^+^ population. Data represent *n* = 5–8 mice per genotype pooled from two independent experiments. Error bars show the mean ± *SEM* and significance was determined by Student's *t*‐test. A *p* < .05 was considered statistically significant (*p < .05, **p < .01)

Subsequently, we analyzed innate‐like B‐1 cells residing in peritoneal cavity and spleen (Figure 2A,B). While peritoneal B‐1 cells were mostly unaltered, we observed a severe reduction of splenic B‐1 cells in CXCR1‐deficient mice, especially affecting the population of CD5^−^ B‐1b cells (Figure 2B). For flow cytometry gating strategies applied to identify corresponding B‐cell populations, the reader is referred to Figure S1.

Since B‐1 cells are well‐established as major producers of circulating IgM under homeostatic conditions, we evaluated the concentration of global serum IgM in these mice. Surprisingly, there were no differences detectable (Figure S2).

### 3.2 Normal antibody response towards TI antigens in CXCR1‐deficient mice

**Figure 3:**
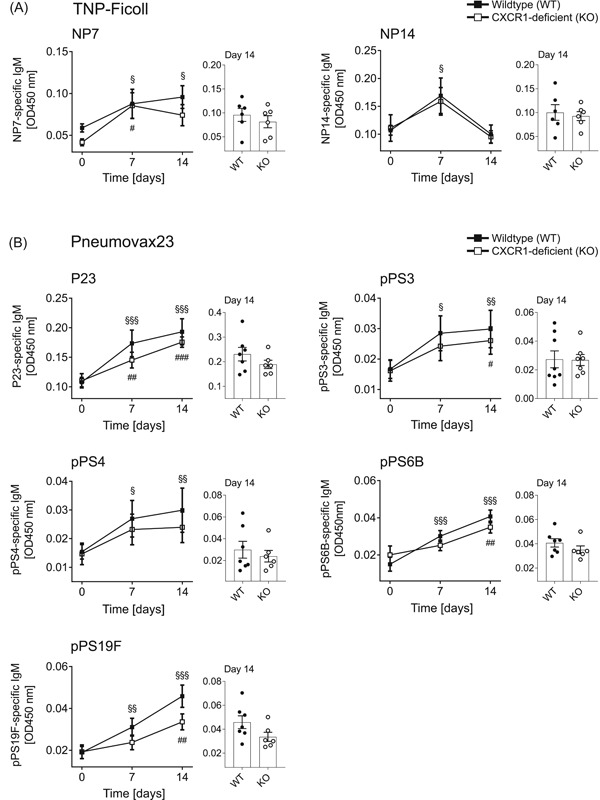
In vivo IgM responses towards the thymus‐independent (TI) antigens TNP‐Ficoll and Pneumovax23 (P23). For analysis of TI immune responses, wild‐type (WT) and CXCR1‐deficient (KO) mice were intraperitoneally immunized with (A) TNP‐Ficoll or (B) P23. Blood sampling was performed before (Day 0) and 7 and 14 days postimmunization to measure NP7 and NP14 (TNP‐Ficoll) or P23, pPS3, 4, 6B and 19F (P23)‐specific antibodies by enzyme‐linked immunosorbent assay (ELISA). Graphs depict the overtime progression of serum IgM levels given as optical density (OD) at 450 nm measurement wavelength. Accessorily, single data points are shown for Day 14. For every specific assay, all probes were analyzed on one identical ELISA plate in duplicates to allow direct comparison of samples. Curves represent *n* = 5–8 mice per genotype out of two independent experiments and error bars illustrate the mean ± *SEM*. Significances were determined by two‐way analysis of variance multiple comparisons and a *p* < .05 was considered statistically significant. ^§^ indicates significance of WT curves and ^#^ of KO curves (^§,#^ *p* < .05, ^§§,##^ *p* < .01, ^§§§,###^ *p* < .001)

Given the diminished pool of splenic B‐1 cells in CXCR1‐deficient mice, we further examined their antibody response towards TI antigens. To this end, mice were immunized with the TI antigen TNP‐Ficoll and blood sampling was performed before, 7 and 14 days after immunization. As depicted in Figure 3A, the induction of specific IgM targeting the epitopes NP7 (left panel) and NP14 (right panel) was equally productive in both, CXCR1‐deficient and WT control mice.

B‐1 cells are known to be essentially involved in the antibody‐mediated response towards pPS. In this regard, they represent an important first line defense but also contribute to long‐lasting immunity against *Streptococcus pneumoniae*.^18^, ^28^, ^29^ Thus, we were interested in the antibody response of CXCR1‐deficient mice towards the pure polysaccharide vaccine P23, provoking TI responses. Figure 3B displays the analysis of specific IgM targeting P23 as well as four common serotypes of *S. pneumoniae*, frequently associated with human disease (pPS3, 4, 6B, and 19F). As illustrated in Figure 3B, the TI antibody production in response to P23‐immunization was unaltered in CXCR1‐deficient mice.

### 3.3 IL‐5/IL‐5Rα‐dependent stimulation of B‐1 cells

**Figure 4:**
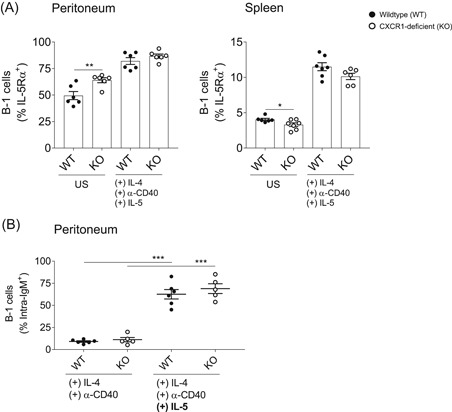
IL‐5/IL‐5Rα‐dependent stimulation of B‐1 cells in vitro. (A) Peritoneal (left panel) and splenic (right panel) cells were isolated and cultured in complete cell medium to investigate the surface expression of IL‐5Rα on B‐1 cells. To this end, 10‐ng/ml IL‐4, 2‐µg/ml α‐CD40, and 10‐ng/ml IL‐5 were supplemented and cells were incubated for 48 h at 37°C and 5% CO_2_. Subsequently, cells were fluorescently stained with CD19‐V450, CD43‐PE, CD5‐APC, IgM‐PE‐Cy7, and CD125/IL‐5Rα‐FITC for flow cytometry. Graphs depict the proportion of IL‐5Rα‐positive B‐1 cells within unstimulated (US) controls and the stimulated cell fraction. (B) Peritoneal cells were stimulated either with IL‐4 and α‐CD40 alone or additionally with IL‐5. Subsequently, cells were stained with CD19‐V450, CD43‐FITC, CD5‐APC, and IgM‐PE on the surface and intracellular with IgM‐PE‐Cy7 for flow cytometry. Graphs illustrate the percentual expression of intracellular IgM in wild‐type (WT) and CXCR1‐deficient (KO) B‐1 cells in the absence or presence of IL‐5. Data comprise *n* = 5–6 mice from each two independent experiments and error bars indicate mean ± *SEM*. Significances were determined by Student's *t*‐test (A) or one‐way analysis of variance multiple comparisons (B) and a *p* < .05 was considered statistically significant (*p < .05, **p < .01, ***p < .001)

The IL‐5/IL‐5R‐signaling pathway is known to be a key regulator of B‐1 cell survival and homeostatic proliferation. IL‐5 binding is mediated through the IL‐5Rα chain, which is constitutively expressed on B‐1 cells.^30^ Accordingly, depletion of the IL‐5Rα chain results in a strong decrease of the B‐1 cell population in mice.^31^ We, therefore, wondered whether the IL‐5 pathway might be affected in the context of CXCR1‐deficiency, leading to the massive reduction in splenic B‐1 cells we could previously observe. Hence, we analyzed IL‐5Rα expression on peritoneal and splenic B‐1 cells before and after in vitro stimulation with IL‐4, α‐CD40, and IL‐5. As illustrated in Figure 4A, the proportion of IL‐5Rα^+^ peritoneal B‐1 cells was significantly increased in the absence of CXCR1 (left panel), while being decreased within the population of splenic B‐1 cells (right panel). Surprisingly, these differences were compensated to a great extent after 48 h of stimulation (Figure 4A).

Besides, IL‐5 efficiently triggers differentiation of peritoneal B‐1 cells into IgM‐producing plasmablasts.^32^ We thus complementarily analyzed the expression of intracellular IgM in peritoneal B‐1 cells after 24 h of stimulation either without or with IL‐5 supplementation. As shown in Figure 4B, intracellular IgM‐expression was specifically induced by the addition of IL‐5 on a highly significant level. There were no differences detected between the genotypes (Figure 4B).

### 3.4 Improved IgG_1_ response towards TD antigen in CXCR1‐deficient mice

As we have found markedly increased populations of splenic B‐2 cells in CXCR1‐deficient mice, we next focused on their antibody response towards TD antigen. To this end, mice were immunized with TNP‐CGG, and antibody reactions were measured at Days 0, 7, and 14. Apart from IgM, we additionally included IgG_1_ and IgG_3_ isotypes in our analysis. Again, we assessed serum Ig levels specifically targeting NP7 (left panel) and NP14 (right panel; Figure 5A). While IgM and IgG_3_ responses were comparable between the genotypes, CXCR1‐deficient mice produced significantly more amounts of NP7‐ and NP14‐specific IgG_1_ at both days postimmunization (Figure 5A).

Figure 5Improved in vivo B‐ and T‐cell responses towards thymus‐dependent (TD) antigen TNP‐CGG in CXCR1‐deficient mice. Wild‐type (WT) and CXCR1‐deficient (KO) mice were immunized intraperitoneally with TNP‐CGG precipitated in alum and blood sampling was performed before (Day 0) and 7 and 14 days after vaccination to measure NP7 and NP14‐specific antibodies by enzyme‐linked immunosorbent assay (ELISA). For every immune globulin (Ig) and specificity, all probes were analyzed on one identical ELISA plate in duplicates to allow for direct comparison. After 14 days, mice were killed and splenocytes were analyzed by flow cytometry. (A) Overtime progression of the amount of specific serum IgM, IgG_1_, and IgG_3_ shown as optical density (OD) at 450 nm measurement wavelength. Curves represent *n* = 7–8 mice per genotype out of two independent experiments and error bars indicate ± *SEM*. Significances were determined by two‐way analysis of variance multiple comparisons and a *p* < 0.05 was considered statistically significant. ^§^ indicates significance of WT curves; ^#^ of KO curves; and * reflect differences between the genotypes (^§,#,^*p < .05, ^§§,##,^**p < .01, ^§§§,###,^***p < 0.001). (B) Splenic germinal center reaction B cells and T_FH_ cells were defined as B220^+^GLY7^+^ and CD3^+^CD4^+^CXCR5^+^, respectively. Results are depicted as rate of all single lymphocytes and percentage within the overall B220^+^ or CD3^+^CD4^+^ T_H_ cell‐population. In addition, the median fluorescent index of CXCR5‐APC is shown for T_H_ cells. Data is shown for *n* = 4–8 mice and error bars represent mean ± *SEM*. Significances were determined by Student's *t*‐test and a *p* < .05 was considered statistically significant (*p < 0.05, **p < .01)

**Figure:**
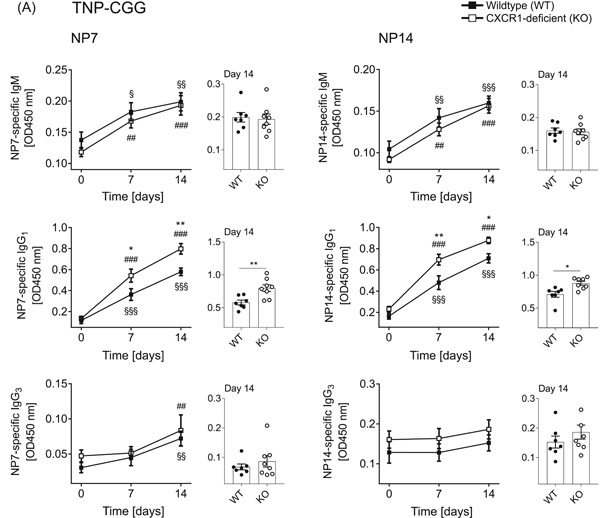

**Figure:**
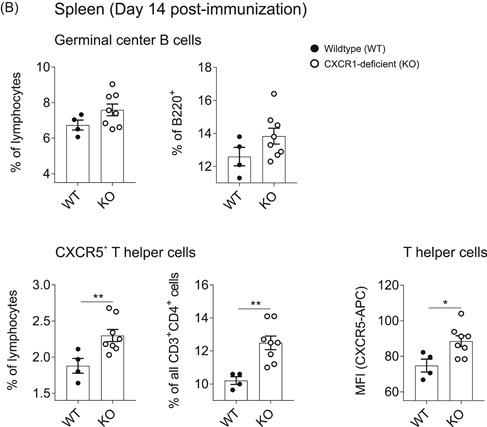

Since TD IgG_1_ antibody responses generally depend on GC reaction B cells in cooperation with CXCR5‐expressing T_H_ cells, we investigated both cell types in the spleen of mice 14 days after TNP‐CGG vaccination.^23^

First, we found the total cell numbers of splenocytes, B220^+^ B cells, CD3^+^ T cells, and CD3^+^CD4^+^ T_H_ cells being similar in both experimental groups (Figure S3A,B). Second, populations of GC reaction B cells did not statistically differ in CXCR1‐deficient mice as compared to their WT counterparts (Figure 5B, upper panel).

However, when analyzing CXCR5‐expressing T_H_ cells, we found that these cells were significantly enriched in the absence of CXCR1 within the overall lymphocyte population as well as within the fraction of all T_H_ cells (Figure 5B, lower panel). These findings were supported by reinforced median fluorescent index of CXCR5‐APC on CD3^+^CD4^+^ T_H_ cells (Figure 3C). Figure S3D exemplarily shows the gating strategy applied for identification of CXCR5^+^ T_H_ cells.

## 4 DISCUSSION

Chemokine receptors and their corresponding chemotactic molecules play a pivotal role in immunity.^33^ While CXCR1 is already established as an important regulator of neutrophil migration and function, its role in the context of antibody responses has not been studied before.

The present work reveals a novel relevance of CXCR1‐expression with regard to TD antibody responses. Upon TNP‐CGG vaccination, we found greatly increased levels of antigen‐specific IgG_1_ in the serum of CXCR1‐deficient mice (Figure 5A). Besides, these mice harbored increased rates of CXCR5^+^ T_H_ cells in the spleen (Figure 5B). The absolute number of the overall T‐cell population was unaltered, indicating that the loss of CXCR1 specifically favored CXCR5‐expression on T_H_ cells (Figures S3B and 5B).

IgG_1_ is the most abundant subclass of the IgG isotype and is classically induced in response to protein antigens.^34^ This involves interactions of B and T cells during GC reaction in the B‐cell follicles.^23^ More precisely, specialized T cells drive the development of high‐affinity GC B cells that eventually differentiate into antibody‐secreting, class‐switched plasma cells.^13^

It is well‐known that activated T_H_ cells need to downregulate CCR7 and at the same time upregulate CXCR5 on their surface to get access to the B‐cell follicle.^35^ Moreover, CXCR5‐expression by T cells is known to increase the probability of direct interaction with their cognate antigen‐specific B cells. Conversely, GC reactions are strongly affected by CXCR5‐deficiency. Among others, its deletion is accompanied by impaired B‐cell isotype class switching.^24^, ^35^, ^36^, ^37^ Based on this knowledge we speculate that increased CXCR5‐expression levels on T_H_ cells might be one mechanism leading to the improved IgG_1_ production in CXCR1‐deficient mice. This might occur through reinforced T‐cell entry into the follicles or by strengthened interactions of T and B cells. Another contributing point might also be the enriched FO B‐cell population in these mice at steady state, being the foundation for enhanced responses after antigen encounter (Figure 1).

Importantly, subsequent studies are needed to clarify whether CXCR1‐expression exerts direct influence on FO B and T_H_ cells, or whether our observations rely on indirect effects. Of note, by performing RT‐PCR, we were able to detect expression of CXCR1 in CD19^+^ splenocytes derived from WT mice, while its expression was absent in CXCR1‐deficient mice. At the same time, the highly homologous receptor CXCR2 was expressed in B cells of both genotypes (Figure S4).

As a consequence, it is reasonable to speculate that CXCR1 expressed by B cells could directly regulate their function. However, to verify this hypothesis, in vitro proliferation and migration assays should be performed to study the role of CXCR1 in B‐cell activation and trafficking. Immune responses are highly versatile and involve the interplay of various cell types. For example, neutrophil function is known to depend on CXCR1 and at the same time, cross‐talk between neutrophils and lymphocytes has been reported.^38^, ^39^ Thus, the increased IgG_1_ response in the context of CXCR1‐deficiency might also rely on inflammatory signals derived from myeloid cells. In summary, our observations are of high interest and CXCR1 should be studied in detail in the context of GC reactions.

Interestingly, the frequency of splenic B‐1b cells was drastically reduced in the absence of CXCR1 (Figure 2A). On the other hand, the enrichment of IL‐5Rα‐expressing cells in the peritoneum of CXCR1‐deficient mice further points to a local accumulation of activated B‐1 cells, which in turn were decreased within the splenic fraction (Figure 4A). Since the divergences in IL‐5Rα‐expression were largely compensated upon stimulation in vitro, these most likely do not result from any functional deficit in the absence of CXCR1. This idea is further supported by the fact that we were unable to detect any differences in natural serum IgM or intracellular IgM expression of stimulated B‐1 cells (Figures S2 and 4, respectively). The latter further indicates that CXCR1 is dispensable for IL‐5‐induced IgM production by peritoneal B‐1 cells (Figure 4B). Moreover, there were no statistically significant differences in antibody production towards TI immunization (Figure 3).

Nevertheless, it is possible that CXCR1‐signaling is involved in the regulation of B‐1 cell distribution under homeostatic conditions. B‐1 cells are characterized by great mobility, as they continuously traffic between the blood and the peritoneal space, passing the omentum.^40^ Moreover, upon activation, they rapidly egress from the peritoneum to enter the spleen.^41^, ^42^ One possibility would be that CXCR1 is required for B‐1 cell homing into the spleen, which would in turn explain the markedly reduced population of splenic B‐1 cells on the background of CXCR1‐deficiency. Strikingly, B‐cell migration and entry into B‐cell follicles is known to involve CXCR5–CXCL13‐mediated signaling and CXCR5 has been shown to be highly expressed on B‐1 cells.^40^ Accordingly, the increased expression of CXCR5 on T_H_ cells in CXCR1‐deficient mice made us wonder whether CXCR5‐expression might also be altered on B‐1 cells. Indeed, we found significantly lower CXCR5‐expression levels on peritoneal B‐1 cells in the absence of CXCR1, while splenic samples were comparable (Figure S5). Among others, homeostatic B‐cell positioning is regulated by CXCR5.^1^ The dampened CXCR5‐expression on B‐1 cells of CXCR1‐deficient might prevent their entry into the spleen, leading to an accumulation of these cells in other locations. However, more data needs to be assessed to support this idea, especially since mechanisms of B‐1 cell trafficking remain poorly understood.

## 5 CONCLUSION

In summary, our data reveals a previously unknown role of CXCR1 during the formation of TD antibody responses in mice. Since we were able to detect expression of CXCR1 in CD19^+^ splenocytes, it is possible that CXCR1 might exert direct influence on B‐cell responses. Moreover, our findings allow the assumption that CXCR1‐mediated signaling might take over a regulatory role for T_H_ cells by controlling CXCR5‐expression levels. These findings could be useful since adaptive antibody responses are commonly hyperactivated in human autoimmune diseases.^43^, ^44^ Besides, the accumulation of CXCR5‐expressing T_H_ cells positively correlates with disease severity in patients suffering from systemic lupus erythematosus, Sjögren's syndrome, and rheumatoid arthritis.^45^, ^46^, ^47^

Based on our novel observations, CXCR1 should be subject to future studies in the context of GC dynamics and B‐cell migration.

## CONFLICT OF INTERESTS

The authors declare that there are no conflict of interests.

## AUTHOR CONTRIBUTIONS

Jennifer Jaufmann designed and carried out the experiments, analyzed the data, and wrote the manuscript. Melanie Carevic performed experiments and provided experimental advice. Derya Eliacik and Leyla Tümen performed experiments. Fee Schmitt designed experiments. Dominik Hartl provided experimental and conceptual advice. Sandra Beer‐Hammer designed experiments, provided experimental and conceptual advice and wrote the manuscript. All authors discussed the data and edited the manuscript.

## Supporting information

Supporting information.

Supporting information.

Supporting information.

Supporting information.

Supporting information.

Supporting information.

## Data Availability

The data that support the findings of this study are available from the corresponding author upon reasonable request.
