# Supplementary figures and images for: Enhanced IgG1‐mediated antibody response towards thymus‐dependent immunization in CXCR1‐deficient mice
Source: Immun Inflamm Dis. 2020 Nov 23;9(1):210–22. doi: 10.1002/iid3.380 (PMC7860589; doi:10.1002/iid3.380)

## B-2 Cell Gating

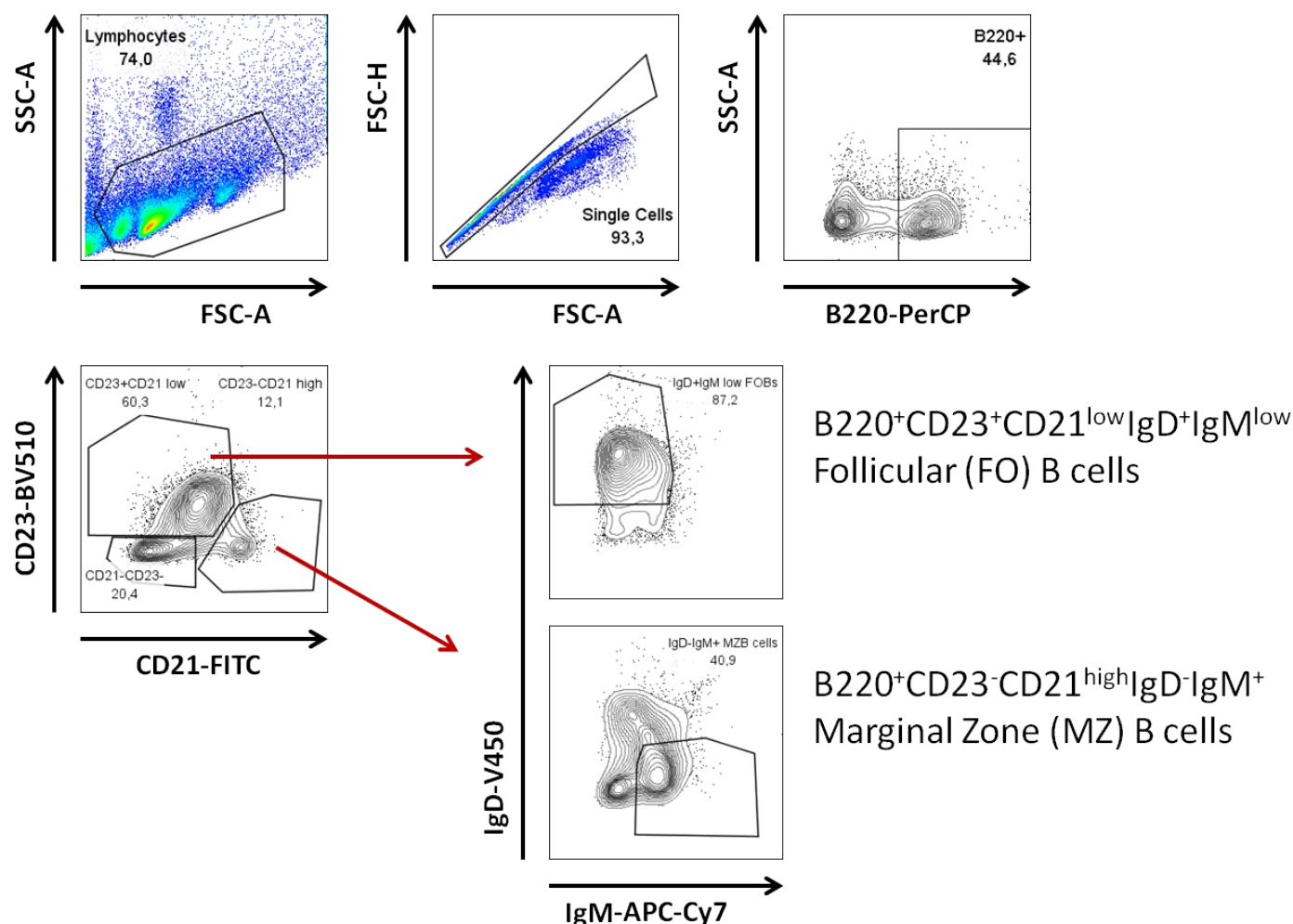

## B-1 Cell Gating

Unstimulated + IL-4,  $\alpha$ -CD40, IL-5

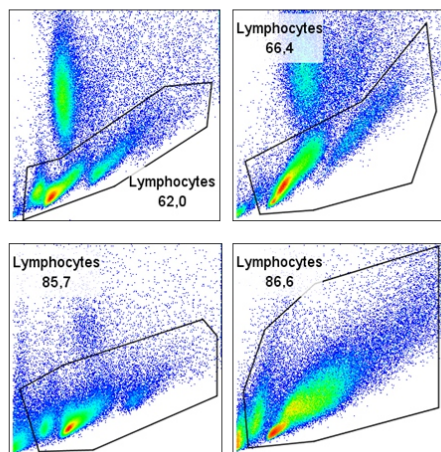

Peritoneum

Spleen

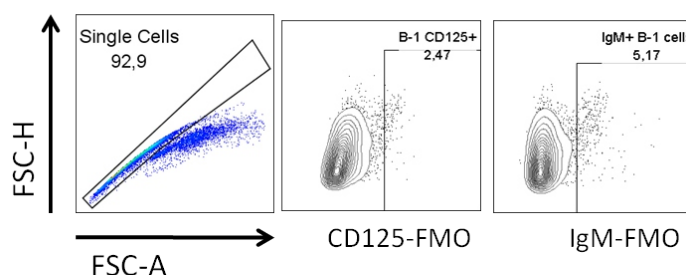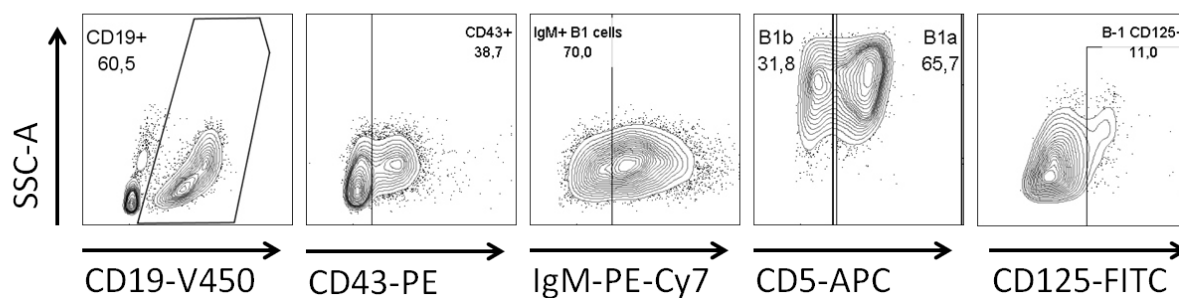

Supplement: Supplementary file 1 — Supporting information. [file IID3-9-210-s001.pdf]

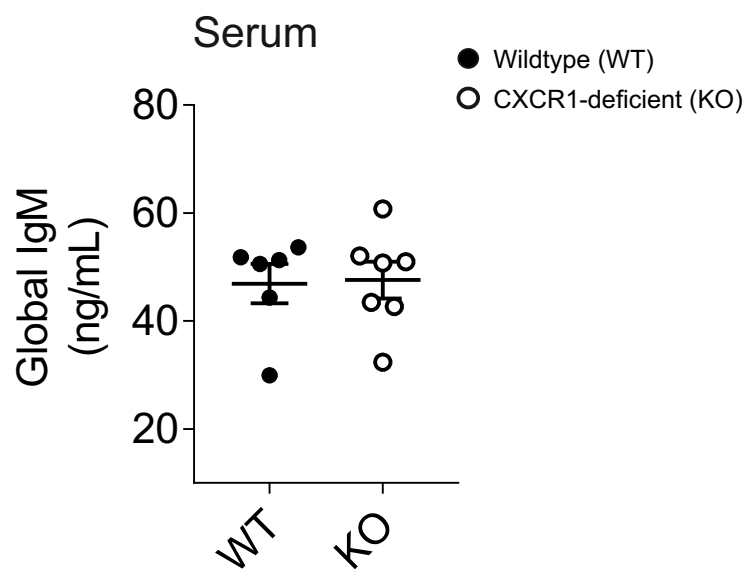

Supplement: Supplementary file 2 — Supporting information. [file IID3-9-210-s002.pdf]

# Spleen (Day 14 post-immunization)

**A**

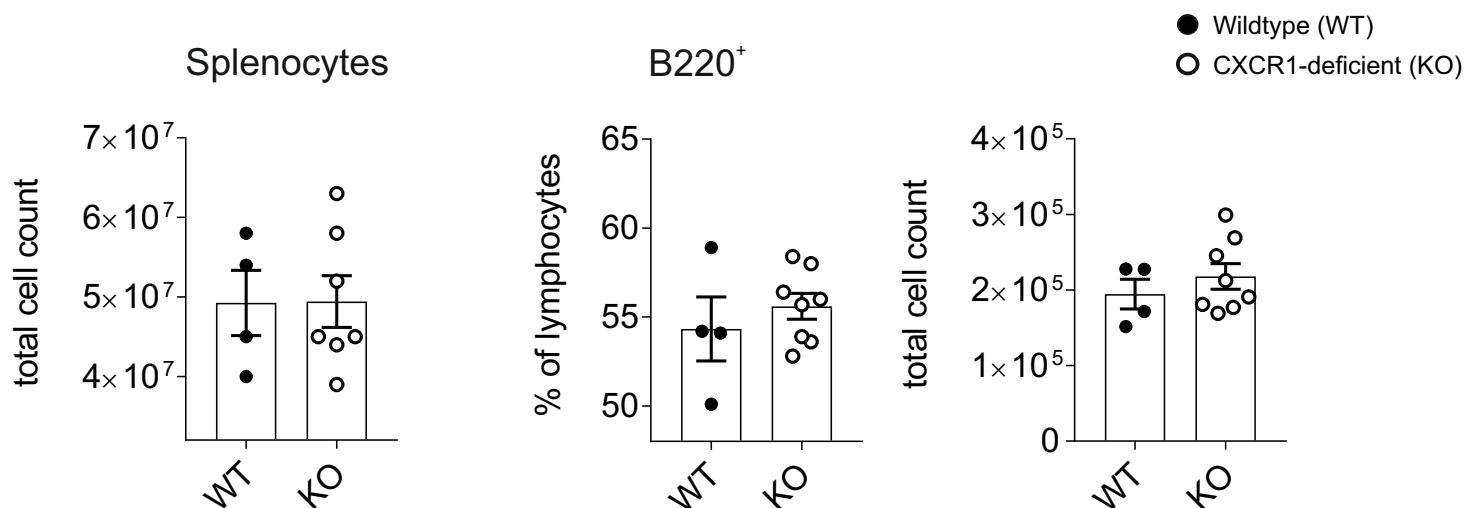

**B**

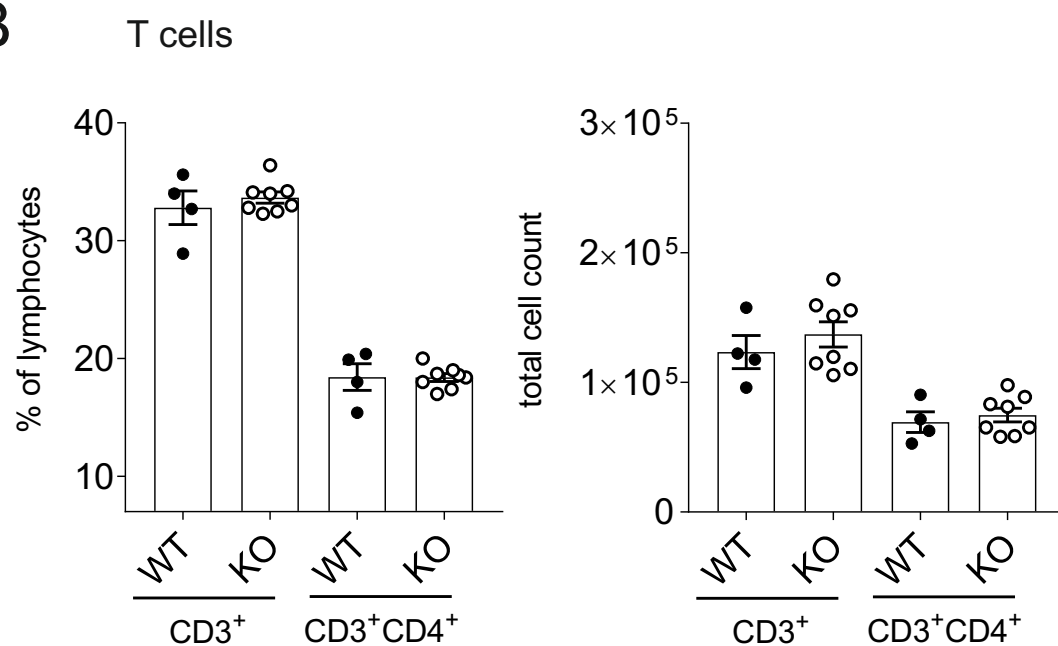

**C**

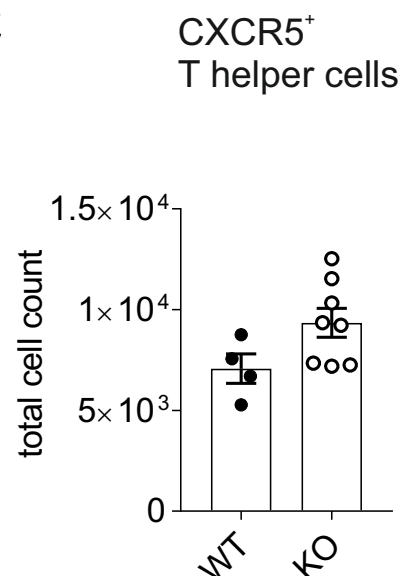

**D**

**T helper cell gating**

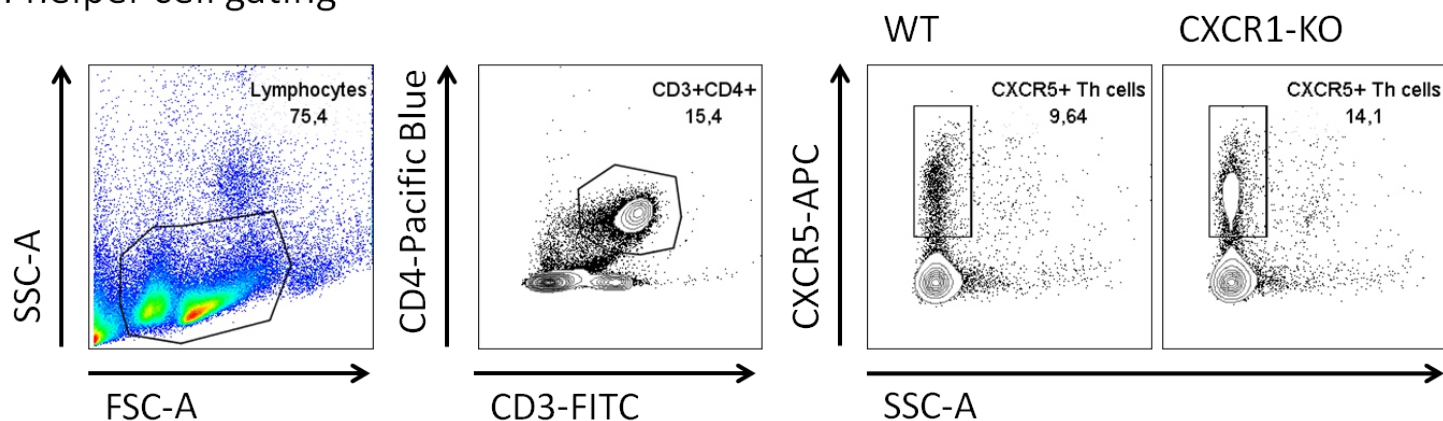

Supplement: Supplementary file 3 — Supporting information. [file IID3-9-210-s003.pdf]

# Expression of CXCR1 and CXCR2 in CD19<sup>+</sup> splenocytes

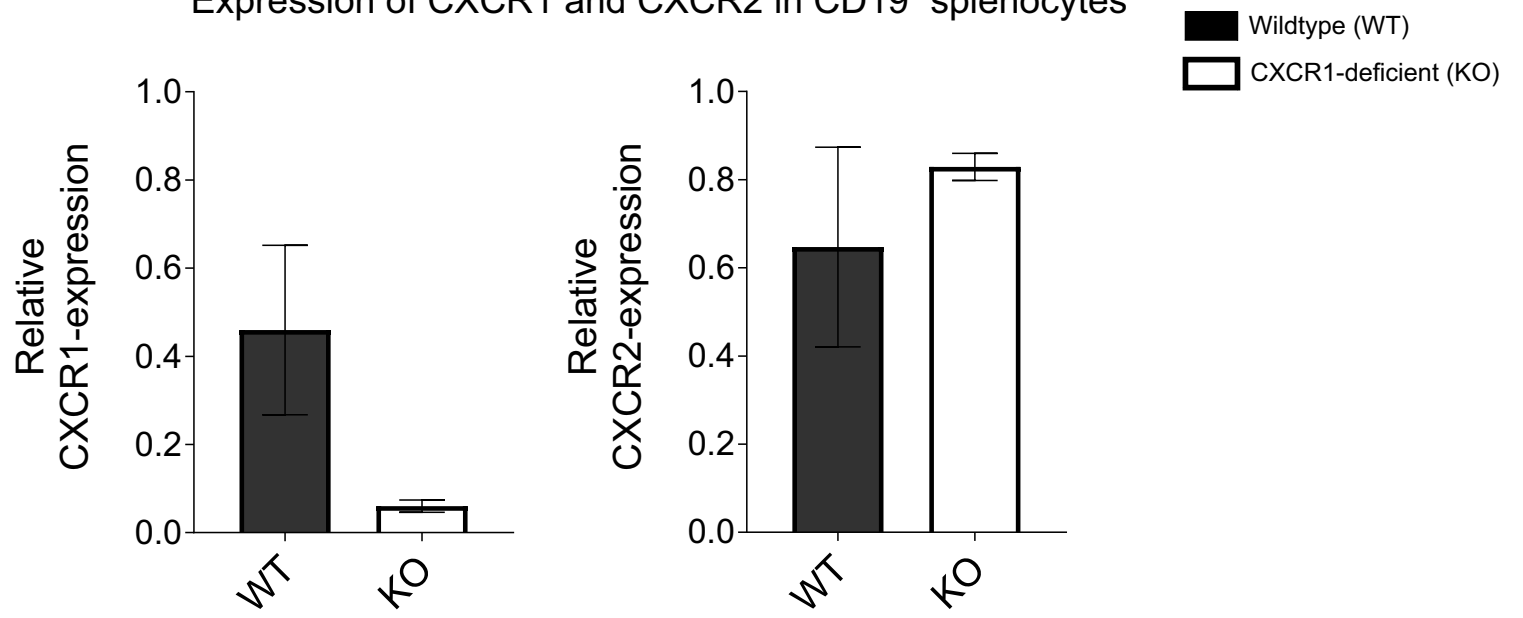

Supplement: Supplementary file 4 — Supporting information. [file IID3-9-210-s004.pdf]

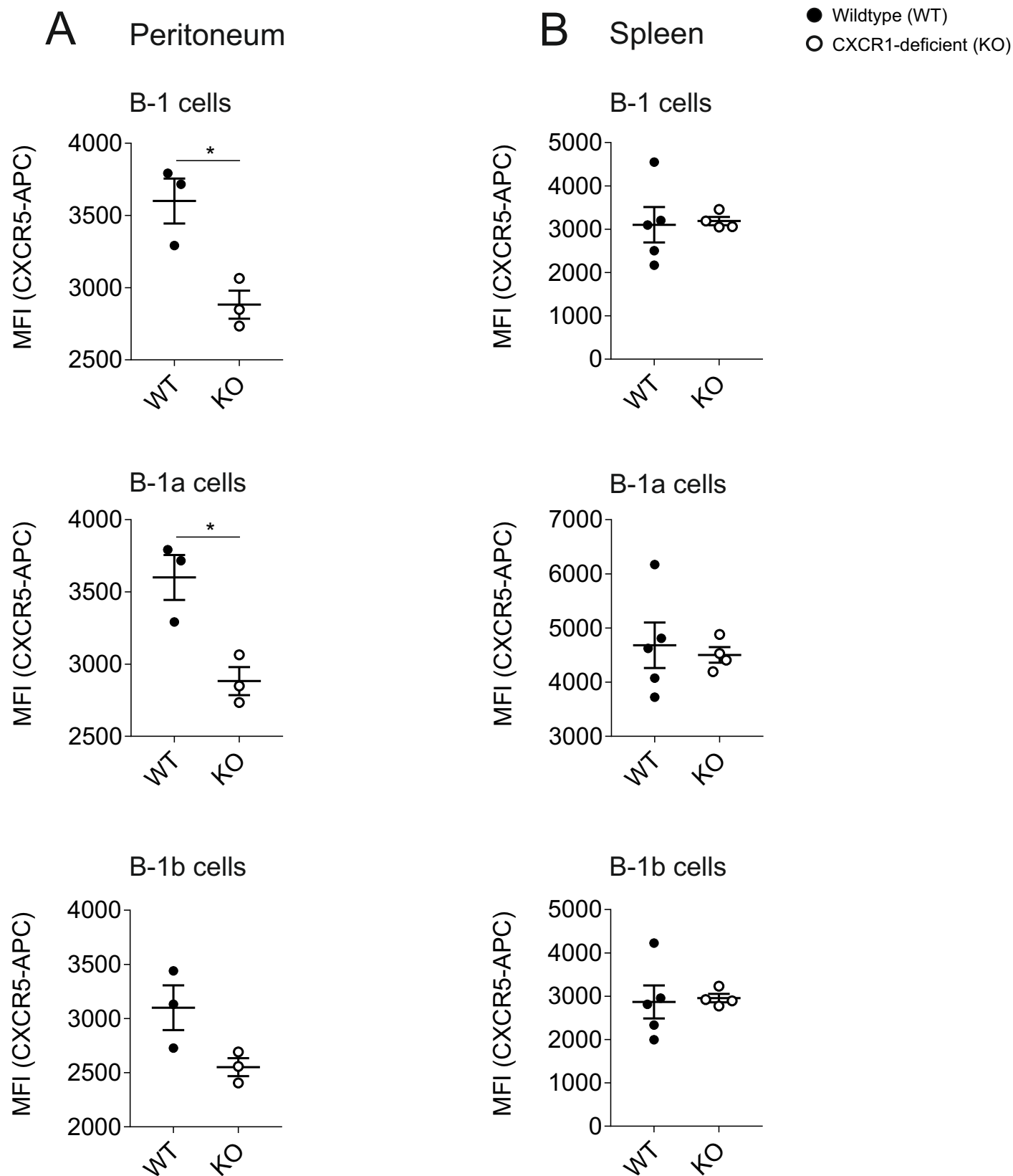

Supplementary Figure 5

Supplement: Supplementary file 5 — Supporting information. [file IID3-9-210-s005.pdf]
