# Supplementary material for: Enhanced IgG1‐mediated antibody response towards thymus‐dependent immunization in CXCR1‐deficient mice
Source: Immun Inflamm Dis. 2020 Nov 23;9(1):210–22. doi: 10.1002/iid3.380 (PMC7860589; doi:10.1002/iid3.380)
Supplement: Supplementary file 6 — Supporting information. [file IID3-9-210-s006.docx]

Supplementary Figure 1: Flow Cytometry Gating Strategies.

Initially, all samples were gated for single lymphocytes. To analyze B-2 cells, B220^+^ cells were selected and divided according to their lack or expression of the surface markers CD23 and CD21. CD23^+^CD21^low^ cells were further gated for IgD^+^IgM^low^ FO B cells, whereas the CD23^-^CD21^high^ population was selected for IgD^-^IgM^+^ MZ B cells. To identify B-1 cells in steady state or post-stimulation, all CD19^+^ cells were positively selected for CD43-expression. Afterwards, all IgM^+^ cells were gated. To define B-1a and B-1b cells, populations were divided into CD5^+/-^. IL-5Rα/CD125-expression was examined within the overall B-1 cell population. For correct definition of the gates, fluorescence minus one (FMO) controls were applied.

Supplementary Figure 2: Global IgM Concentrations in the Sera of Mice at steady State.

Concentrations of serum IgM in wildtype (WT) and CXCR1-deficient (KO) mice were determined by ELISA at day 0 and are given in ng/mL as calculated by linear standard curve. Graph depicts n=6-7 mice per genotype from at least two independent experiments and error bars indicate mean ± SEM.

Supplementary Figure 3: Immune Cell Populations of the Spleen 14 Days post-Immunization with TNP-CGG.

14 days after vaccination with TNP-CGG, all mice were sacrificed and spleens were homogenized and analyzed by flow cytometry. Graphs illustrate **(A)** the total cell count of splenocytes and both, percentage and total cell count of B220^+^ cells, **(B)** T-cell population frequencies within the fraction of all single lymphocytes and total T-cell counts, **(C)** the total cell count of CD3^+^CD4^+^CXCR5^+^ T_FH_ cells. n=4-8 wildtype (WT) and CXCR1-deficient (KO) mice were analyzed and error bars depict mean ± SEM and **(D)** an exemplary gating strategy of CXCR5^+^ T_FH_ cells.

Supplementary Figure 4: Expression of CXCR1 and CXCR2 in CD19^+^ splenocytes.

To analyze the expression of CXCR1 and 2 in B cells, CD19^+^ splenocytes were enriched via MACS. Subsequently, total RNA-contents were isolated and transcribed into cDNA, followed by performance of RT-PCR. ß-actin served as reference gene for normalization. The graphs depict the mean ± SEM of relative CXCR1-expression (left panel) and CXCR2-expression (right panel) in CD19^+^ cells from n=2 wildtype (WT) and n=3 CXCR1-deficient (KO) animals.

Supplementary Figure 5: CXCR5-Expression on B-1 Cells of Peritoneum and Spleen.

To assess CXCR5-expression levels on B-1 cells via flow cytometry, isolated single cell suspensions from wildtype (WT) and CXCR1-deficient (KO) mice were stained with CD19-FITC, CD43-PE-Cy7, CXCR5-APC, IgM-APC-Cy7 and CD5-PE. Graphs illustrate the median fluorescent index (MFI) of CXCR5-APC expression on the CD19^+^CD43^+^IgM^+^ B-1 cell population of **(A)** the peritoneum and **(B)** the spleen. Data represents n=3-5 mice of each genotype and error bars depict the mean ± SEM. Significance was determined using *Student’s t-test* and a p-value of <0.05 was considered statistically significant (p*<0.05).
